# Implementing Carotid Ultrasonography in Optimizing Primary Cardiovascular Prevention Strategy: Has the Time Come?

**DOI:** 10.3390/jcm12062193

**Published:** 2023-03-12

**Authors:** Anastasios Kollias, Konstantinos G. Kyriakoulis, Panagiota Stathopoulou, George Stergiou

**Affiliations:** Hypertension Center STRIDE-7, Third Department of Medicine, Sotiria Hospital, School of Medicine, National and Kapodistrian University of Athens, 11527 Athens, Greece; konkyriakoulis@gmail.com (K.G.K.);

The cardiovascular (CV) disease continuum begins from a cluster of CV risk factors, proceeds with the development of asymptomatic atherosclerotic lesions and ends with the occurrence of CV events. The prediction of the CV risk is of paramount importance for defining the optimal primary prevention strategy in individual patients, in terms of prompt initiation of treatment for all the modifiable CV risk factors, choice of indicated drug categories, and optimal treatment targets. 

The total CV risk assessment is routinely performed by using several CV risk calculating models [1]. However, despite the improvements in the CV risk calculators, these provide predictions only at a certain statistical level with an ‘acceptable’ margin of error, whereas there are several limitations such as the dynamic variation in the levels of the systolic blood pressure [2], the inability to incorporate the duration of the previous exposure to a risk factor (i.e., a high smoking load, or a delayed initiation of antihypertensive and/or hypolipidemic treatment), the inability to distinguish whether normal values of a risk factor are due to current drug treatment, the presence of other important risk-modifiers that are not considered, etc. Furthermore, even in the case that the risk calculators would be highly accurate at an individualized level, the heterogeneity in the recommendations by different societies based on these risk equations renders the situation rather problematic for clinical application.

The prompt identification of the asymptomatic atherosclerotic lesions is crucial for the reclassification of a low/moderate CV risk patient to the category of high or very high CV risk and for the optimal implementation of an aggressive treatment strategy aiming at preventing future CV events [3]. Among various tests designed to evaluate the indices of asymptomatic target-organ damage with potential reclassification value, carotid ultrasonography has been the focus of clinical interest and intense research [4,5]. Carotid ultrasonography allows the assessment of both structural (atherosclerotic plaque burden) and functional (local stiffness) arterial wall properties and several indices including carotid intima-media thickness, carotid distensibility, and carotid plaque characteristics have been all associated with increased CV risk [4,5,6,7,8,9]. However, the presence of carotid plaque appears to have the strongest prognostic value in terms of CV risk reclassification [4,8]. Further improvement in risk estimation may be gained by considering not only the presence of the carotid plaque, but also other characteristics, such as plaque echogenicity and heterogeneity (intraplaque hemorrhage), surface irregularities, neovascularization, size (volume and area, total plaque score), which appear to carry additional adverse prognostic value [4,5]. 

A recent meta-analysis of studies conducted in the general population aged 30–79 years showed that the global prevalence of increased carotid intima-media thickness, carotid plaque, and significant stenosis (≥50%) is estimated at 27.6%, 21.1%, and 1.5%, respectively [10]. Thus, carotid atherosclerosis is present in at least 1 out of 4 or 5 individuals in the general population, although severe lesions are rare. Current guidelines recommend against routine screening for asymptomatic carotid artery stenosis in the general adult population [11]. This recommendation is certainly valid when the endpoint of interest regards the identification of severe stenosis which is eligible for surgical intervention. On the other hand, the current guidelines acknowledge that the identification of carotid plaques reclassifies patients from intermediate to high CV risk, or to very high CV risk in case of advanced stenosis (≥50%) [3]. Thus, the implementation of vascular ultrasonography in individuals at moderate CV risk may have serious therapeutic implications, as it may reclassify an important proportion of them in whom the total risk has been underestimated [12]. 

The case of dyslipidemia management reflects the difficulties in the definition of the optimal treatment strategy based on CV risk assessment models. The Systematic COronary Risk Evaluation-2 (SCORE2) is an updated algorithm calibrated and validated to predict the 10-year risk of first-onset CV disease in European populations with different region-specific CV risk levels [1]. The 2021 European guidelines on CV disease prevention used SCORE2, but dramatically reduced eligibility for a class I recommendation for statin therapy in low CV risk countries to only 4% of individuals, aged 40–69 years, and less than 1% of women [13,14]. This was in contrast to the previous 2019 European guidelines, as well as current UK-National Institute for Health and Care Excellence and US-American College of Cardiology/American Heart Association guidelines, that provided class I/strong recommendations to 20%, 26%, and 34% of individuals, respectively [13,14]. In a recent study among individuals without established CV disease, implementation of carotid ultrasonography and identification of carotid plaque considerably increased hypolipidemic treatment eligibility more evidently in women than men (from 11% to 71% for women and from 26% to 61% for men) [15].

Carotid ultrasonography allows the direct visualization and assessment of the atherosclerotic burden in a window of the arterial bed, whereas at the same time is a convenient noninvasive examination easily accepted by patients. However, the standardization of performing carotid ultrasonography, and most importantly reporting and interpreting the main findings is crucial for clinical decision making. Clinicians dealing with CV risk management should consider implementing carotid ultrasonography in individuals with moderate CV risk who may be eligible for drug treatment. Close collaboration with radiologists or even training in performing basic scanning for plaques—even with portable handheld and easy-to-use devices—would contribute to wider and more precise CV risk assessment in routine clinical practice.
